# Determinants of access to and use of maternal health care services in the Eastern Cape, South Africa: a quantitative and qualitative investigation

**DOI:** 10.1186/1756-0500-7-723

**Published:** 2014-10-15

**Authors:** Mluleki Tsawe, Appunni Sathiya Susuman

**Affiliations:** Department of Statistics & Population Studies, University of the Western Cape, Cape Town, South Africa

**Keywords:** Access, Antenatal care, Public health, Maternal health, Primary health care, Health facility, Mdantsane

## Abstract

**Background:**

The main aim of the study is to examine whether women in Mdantsane are accessing and using maternal health care services. Accessibility of maternal health care facilities is important in ensuring that lives are saved through the provision and use of essential maternal services. Therefore, access to these health care services directly translates to use – that is, if women cannot access life-saving maternal health care services, then use of such services will be limited.

**Findings:**

The study makes use of mixed methods to explore the main factors associated with access to and use of maternal health care services in Mdantsane. For the quantitative approach, we collected data using a structured questionnaire. A sample of 267 participants was selected from health facilities within the Mdantsane area. We analyzed this data using bivariate and multivariate models. For the qualitative approach, we collected data from health care professionals (including nurses, doctors, and maternal health specialists) using one-on-one interviews. The study found that women who were aged 35–39, were not married, had secondary education, were government employees, and who had to travel less than 20 km to get to hospital were more likely to access maternal health services. The qualitative analysis provided the insights of health care professionals regarding the determinants of maternal health care use. Staff shortages, financial problems, and lack of knowledge about maternal health care services as well as about the importance of these services were among the major themes of the qualitative analysis.

**Conclusion:**

A number of strategies could play a big role in campaigning for better access to and use of maternal health services, especially in rural areas. These strategies could include (a) the inclusion of the media in terms of broadcasting information relating to maternal health services and the importance of such services, (b) educational programs aimed at enhancing the literacy skills of women (especially in rural areas), (c) implementing better policies that are aimed at shaping the livelihoods of women, and (d) implementing better delivery of maternal health care services in rural settings.

## Background

### The South African health-care perspective

Primary health care (PHC) is an initiative implemented by the South African government to provide basic health care within the public health sector. Several developing countries have initiated this type of health care system as a means of delivering health care services to the populations which cannot afford the services provided by the private health sector [1–3]. Even though primary health care is offered free of charge, there are still people who do not adequately utilize the available health services. In South Africa, as with other developing countries, the primary health care system encounters many challenges – with regards to ‘management’, health ‘coverage’, and negative perceptions of the population based on treatment received at the facilities [1–3]. This has resulted in the health system being dubbed as one that is constantly in a crisis by media reports. Some health care facilities are not adequately equipped to handle the health care needs of their populations (especially in rural areas) and some of these facilities do not provide the health services that are required of them. All of these shortcomings have the potential of contributing to less (or minimal) usage of health care services [4, 5].

The PHC sector is often surrounded by much debate, more so when it comes to the issue of resources and availability of facilities, particularly in rural areas. Health resources are often unequally “distributed among rural and urban areas”. including the unequal distribution of health facilities [6]. This uneven distribution presents barriers mainly when it comes to health care use in both urban and rural areas. Health facility availability and accessibility are some of the major determinants of health care use, even more so for most of the rural areas of South Africa [7–9] – where the provision of health facilities closer to the people is often neglected by the relevant stakeholders.

Most of the challenges within the primary health care sector are attributed to the apartheid regime, which is argued to have laid the backdrop for the ‘failing’ and ‘inadequate’ primary health care system [10]. Therefore, the current state of health care is often viewed in relation to the apartheid regime’s unequal health care provision among the country’s different population groups. The apartheid government was all about rule and divide, which meant that health services were unequally distributed across the population groups [11, 12]. Although the South African government has made strides in promoting equal health care across all population groups since the advent of democracy, there is a lot that needs to be done with regards to providing equal health care among people of differing socio-economic statuses [13]. It is, therefore, necessary for the government (and the country as a whole) to find strategies that will promote health care usage and focus more on the future rather than on the past.

### Maternal health care in the South African context

In South Africa, maternal health care is offered through the primary health care sector – for the majority of the population. As noted above, this health sector is often surrounded confronted with several shortcomings which inhibit the access and use of maternal health care services by the population. This also reflects in the frequency of use of antenatal care, delivery care, postnatal care, as well as other related health services. In addition, improving maternal health is one of the millennium development goals that South Africa as well as many developing countries has to achieve by 2015. It seems less likely that this country will achieve this goal in less than two years. The World Health Organization requires that all women have access to (and utilize) effective maternal health services [14]. Some health facilities in the country do not have the adequate health standards to meet the requirements as set out by the World Health Organization. Moreover, the services and personnel at some of the public health institutions hinder the effective utilization of maternal health services [15]. Most people who use the public health system have had some bad experience which is a factor in the lack of trust in the services provided by this system [16].

#### Accessibility and other barriers to use of maternal health care (and general health) services

Many factors play a role in this inadequate use of maternal health care services such as: lack of information, cultural factors, and educational attainment of the women especially among those residing in rural areas [17]. Accessibility of maternal health care facilities and general health facilities is important in ensuring that lives are saved through the provision of essential maternal (or medical) services. Access to health care services directly translates to use of these services – meaning that, if people cannot access life-saving health care services, then use of such services will be limited. Distance (or travelling time) to health care facilities is one of the major barriers to health care use, more especially in rural South Africa, where health care centers are often located further away from a large number of residents [6, 18]. In order to receive adequate health care, rural residents and some few urban residents, have to travel long distances, and they also have to wait in long queues before being assisted by health care personnel who are often disrespectful and show a non-caring attitude [16, 19].

The rural context of the country presents the greatest challenge to the government because most households and locations are scattered far apart from each other. Women in rural settings tend to use maternal health services far less than women in urban settings [20] – which could be explained by the fact that health care resources are often unequally distributed between rural and urban areas.

### Aim of study

The specific aim of this study is to examine whether women in Mdantsane are accessing and using maternal health care services. Therefore, the general objective is to explore the factors associated with access to and use of maternal health care services.

### Data and methods

The research and data collection involved randomly selecting participants at certain hospitals in the Eastern Cape, South Africa. Participants were drawn from Mdantsane and the surrounding rural areas. The researchers verbally explained the focus of the study and its aims, and also explained the importance of the study. Ethical considerations (such as anonymity, confidentiality, as well as informed consent) were also explained. The researchers made use of verbal informed consent; the main reason for using this form of informed consent was that some of the women (participants) did not know how to read and some did not know how to write. This was done so that all the participants would be on the same level regarding their participation in the study; hence we opted for an all-inclusive approach of informed consent.

Upon gaining access to the participants, we realised that most of them were hesitant to participate in the study due to the forms that we had prepared for them to sign. One of them even asked, “Why should she sign her name if the study participants will be anonymous as we had explained?” Due to finding out that most women did not want to participate if they were asked to sign a page giving their informed consent, we therefore decided that it would be best to get verbal informed consent by checking with the ethics committee governing our research. The participants were further informed that their details were not going to be made available to anyone using the data; their anonymity was thus protected. Our ethical statement is that there is no way to link the responses to them, hence the study is completely anonymous.

The procedures and consent process for this study were reviewed and approved by the Senate Higher Degrees (SHD) via the Faculty’s Post Graduate Committee, at the University of the Western Cape. In accordance with the protocol, a comprehensive informed consent process was followed and no names or identifying information were recorded. Eligible respondents who provided verbal consent to participate were interviewed. The researchers explained the consent form in the presence of a peer educator who was working with the organization implementing the maternal health program in the area. We also signed the consent form after taking (verbally) the consent of the participant (to prove its completion). These forms were stored in a location which was accessible only to the principal investigators of the study. Further, interviews were conducted in locations where women were comfortable and their privacy was assured.

#### Data

The data used are primary data, which were obtained from 267 female participants and six health workers using both quantitative as well as qualitative data collection methods. The quantitative data are analysed using SPSS (Statistical Package for the Social Sciences) version 16.0.

## Methods

### Qualitative investigation phase

The qualitative approach involved collecting data from health care professionals, using one-on-one interviews. A range of questions were asked to gain the health professionals’ views on certain maternal health care problems which they encounter while working at the health facilities, as well as some of the barriers that hinder women from utilizing such services. The qualitative analysis involved selecting main themes from the responses of the interviewees and exploring those. For the analysis and interpretation, responses from two health professionals (out of each of the different professions – i.e. two doctors, two nurses, and two maternal health care specialists) were selected.

### Quantitative investigation phase

The quantitative approach involved analysing data using bivariate and multivariate models. The participants were asked a range of questions, in a structured questionnaire format, regarding their access to maternal health care services and utilization of these services. Below are the variables that we selected for the study.

### Selected variables

We selected variables that were aimed at exploring the determinants of access to and use of maternal health services in the study area. For that reason, all the variables that were selected for the bivariate analysis were also included in the regression model. These variables are thus listed below.

The dependent variables included: (a) access to maternal health services, which had dichotomous categories (0 = no; 1 = yes), and (b) antenatal visits for pregnancy, which had dichotomous categories (0 = 1 to 4; 1 = 5+).

The independent variables included were: age (grouped into six categories: 15-19; 20-24; 25-29; 30-34; 35-39; 40 and above); marital status (dichotomous categories: 1 = married; 2 = not married); maternal education (grouped into four categories: none; primary; secondary; tertiary); occupation (grouped into six categories: none; housewife; self-employed; government employee; private employee; living on social grants); residential area (dichotomous categories: 1 = within Mdantsane; 2 = outside Mdantsane); distance to hospital/health facility (dichotomous categories: 1 = more than 20 km; 2 = less than 20 km); financial difficulties (grouped into three categories: difficult; not difficult; did not try to find/get money); means of transport (grouped into three categories: walk; public transport; private transport); cultural factors (dichotomous categories: 1 = yes; 2 = no); did you find the health care information useful? (dichotomous categories: 1 = yes; 2 = no); knowledge of antenatal services offered (dichotomous categories: 1 = yes; 2 = no); and medical aid (dichotomous categories: 1 = yes; 2 = no).

## Findings

### Quantitative analysis

#### Access to maternal health services

Table 1 presents results from the bivariate analysis regarding access to maternal health services and use of antenatal services. Just over a third (35.2%) of the women surveyed were accessing maternal health services. Factors which are significantly associated with access to maternal health services include occupation (P < 0.05), cultural factors (P < 0.001), finding the information provided useful (P < 0.001), and having access to medical aid (P < 0.001). The profile by age shows that women aged 15–39 accessed maternal health services more compared with those aged 40 and above. Only 23.1% of women aged 40 and above accessed maternal health services. Regarding maternal education, women with no education and those with secondary education had higher proportions regarding access to maternal health services; with those with primary education being the lowest (28.6%). Being a government employee (with a proportion of 87.5%) gave women a better chance of having access to maternal health services. The distance that women had to travel to their health facility also determined the frequency with which they accessed maternal health services, whereby those who reported that they travel less than 20 km to reach their health facility accessed these services at a higher rate (37.7%) compared with those who reported that they travel more than 20 km. Another determining factor is that those who had no financial constraints (42.2%) accessed maternal services more than those who reported that they did not even try to find/get money to travel to hospital (18.2%). Women who reported that their means of traveling to hospital was walking had higher proportions (44.4%) regarding access to maternal health services. This could perhaps be a reflection of the facilities being nearby to those who walked to hospital. Women who reported that they found the information provided within the maternal health care setting useful accessed maternal health services more (58.8%) compared with those who did not find the information useful (27.1%). A similar trend can also be noticed when it comes to having medical aid. Women who have medical aid, accessed maternal health services more (89.5%) than women who do not have medical aid (31.0%).

**Table 1:** **Distribution of background characteristics associated with access to and use of maternal health services in Mdantsane (with** ***p-values*** **and** ***chi-square*** **)**

Characteristics	Access to maternal health services				Antenatal visits for pregnancy			
	No	Yes	P-value	χ^2^	1 to 4	5+	P-value	χ^2^
	(n =173)	(n =94)			(n =156)	(n =111)		
***Age***			0.8	2.2			0.0**	14.3
15-19	61.5	38.5			69.2	30.8		
20-24	66.0	34.0			75.5	24.5		
25-29	62.9	37.1			62.9	37.1		
30-34	64.1	35.9			54.7	45.3		
35-39	61.2	38.8			44.9	55.1		
40 and above	76.9	23.1			42.3	57.7		
***Marital status***			0.9	0.0			0.8	0.1
Married	65.5	34.5			60.0	40.0		
Not married	64.6	35.4			58.0	42.0		
***Maternal education***			0.7	1.4			0.6	2.1
None	62.5	37.5			75.0	25.0		
Primary	71.4	28.6			55.1	44.9		
Secondary	62.8	37.2			57.4	42.6		
Tertiary	68.2	31.8			68.2	31.8		
***Occupation***			0.0***	13.5			0.2	6.8
None	61.1	38.9			52.8	47.2		
Housewife	80.0	20.0			53.3	46.7		
Self-employed	69.2	30.8			74.4	25.6		
Government employee	12.5	87.5			50.0	50.0		
Private employee	74.3	25.7			65.7	34.3		
Living on social grants	66.1	33.9			56.5	43.5		
***Residential area***			0.3	1.2			0.5	0.4
Within Mdantsane	62.3	37.7			56.9	43.1		
Outside Mdantsane	69.0	31.0			61.0	39.0		
***Distance to hospital/health facility***			0.3	1.0			0.6	0.2
More than 20 km	68.1	31.9			60.2	39.8		
Less than 20 km	62.3	37.7			57.1	42.9		
***Financial difficulties***			0.2	2.9			0.6	0.9
Difficult	66.1	33.9			58.3	41.7		
Not difficult	57.8	42.2			60.9	39.1		
Did not try to find/get money	81.8	18.2			45.5	54.5		
***Means of travelling/transport***			0.2	3.4			0.2	3.4
Walk	55.6	44.4			38.9	61.1		
Public transport	64.1	35.9			59.3	40.7		
Private transport	83.3	16.7			66.7	33.3		
***Cultural factors***			0.0*	8.7			0.6	0.4
Yes	25.0	75.0			66.7	33.3		
No	66.7	33.3			58.0	42.0		
***Did you find the health care information useful?***			0.0*	22.3			0.8	0.0
Yes	41.2	58.8			57.4	42.6		
No	72.9	27.1			58.8	41.2		
***Knowledge of maternal services offered***			0.1	3.6			0.9	0.0
Yes	62.3	37.7			58.3	41.7		
No	77.3	22.7			59.1	40.9		
***Medical aid***			0.0*	26.4			0.6	0.3
Yes	10.5	89.5			52.6	47.4		
No	69.0	31.0			58.9	41.1		

Note: 5+ = 5 and above; Significance level: * = P<0.001; ** = P<0.01; *** = P<0.05.

The multivariate analysis (Table 2) showed that women aged 35–39 were 2.0 times more likely to access maternal health services compared with those aged 15–19. Those women who reported that they are not married were (0.7 times) less likely to access maternal health services than those who are married. Regarding maternal education, women with secondary education were 1.8 times more likely to access maternal health services than those with no education. Government employees were 5.0 times more likely to access maternal health services than those who are unemployed. Women who travel less than 20 km to get to hospital were 2.7 times more likely to access maternal health services than women who travel more than 20 km. Regarding financial constraints, women who reported that they do not find it difficult to get money to travel to hospital were 1.6 times more likely to access maternal health services compared with those who found it difficult. Women who reported that they used private transportation to travel to hospital, those who did not find the maternal health services offered useful, those who do not have medical aid, and those who had no knowledge of the services offered were less likely to access maternal health services.

**Table 2:** **Logistic regression model showing the β coefficient and odds ratios of factors associated with access to maternal health services and use of antenatal services in Mdantsane**

Characteristics	Access to maternal health services				Antenatal visits for pregnancy			
	Model I				Model II			
	β	Odds ratio	95% C.I.		β	Odds ratio	95% C.I.	
			Lower	Upper			Lower	Upper
***Age***								
15-19®		1				1		
20-24	0.4	1.5	0.3	6.9	−0.2	0.9	0.2	3.5
25-29	0.4	1.5	0.3	6.8	0.7	2.0	0.5	7.6
30-34	0.2	1.2	0.3	5.7	1.0	2.7	0.7	10.6
35-39	0.7	2.0	0.4	10.2	1.7	5.5***	1.3	23.4
40 and above	0.2	1.2	0.2	7.2	1.7	5.3***	1.1	25.7
***Marital status***								
Married®		1				1		
Not married	−0.3	0.7	0.3	1.8	0.4	1.5	0.7	3.2
***Maternal education***								
None®		1				1		
Primary	0.1	1.1	0.2	8.0	0.6	1.9	0.3	13.9
Secondary	0.6	1.8	0.3	10.6	1.1	2.9	0.4	19.1
Tertiary	0.2	1.2	0.2	10.3	0.5	1.6	0.2	14.2
***Occupation***								
None®		1				1		
Housewife	−1.3	0.3	0.1	1.6	−0.6	0.6	0.2	2.1
Self-employed	−0.6	0.6	0.2	1.4	−1.2	0.3**	0.1	0.7
Government employee	1.6	5.0	0.4	57.9	−0.1	0.9	0.2	4.9
Private employee	−1.3	0.3***	0.1	0.9	−0.5	0.6	0.2	1.5
Living on social grants	−0.4	0.7	0.3	1.5	−0.4	0.7	0.3	1.4
***Residential area***								
Within Mdantsane®		1				1		
Outside Mdantsane	0.3	1.3	0.4	4.2	−0.3	0.8	0.3	1.9
***Distance to hospital/health facility***								
More than 20 km®		1				1		
Less than 20 km	1.0	2.7	0.9	8.7	−0.3	0.8	0.3	1.9
***Financial difficulties***								
Difficult®		1				1		
Not difficult	0.4	1.6	0.7	3.4	0.1	1.1	0.6	2.3
Did not try to find/get money	−1.3	0.3	0.0	8.2	0.7	2.1	0.4	10.9
***Means of travelling/transport***								
Walk®		1				1		
Public transport	−0.4	0.7	0.2	2.3	−0.9	0.4	0.1	1.3
Private transport	−1.9	0.2	0.0	1.4	−1.4	0.2	0.0	1.2
***Cultural factors***								
Yes®		1				1		
No	−2.3	0.1*	0.0	0.5	0.5	1.7	0.4	6.4
***Did you find the health care information useful?***								
Yes®		1				1		
No	−1.3	0.3*	0.1	0.5	−0.2	0.8	0.4	1.6
***Knowledge of maternal services offered***								
Yes®		1				1		
No	−1.3	0.3***	0.1	0.8	−0.1	0.9	0.4	1.9
***Medical aid***								
Yes®		1				1		
No	−3.4	0.0*	0.0	0.2	0.0	1.0	0.3	3.3
**Constant**	**5.3**	**190.9**			**−1.4**	**0.3**		

Note: Significance level: * = P<0.001; ** = P<0.01; *** = P<0.05; 5+ = 5 and above.

® = Reference category.

#### Use of antenatal services

More than half (58.4%) of the women went for at least four antenatal visits, while 41.6% went for five and more antenatal visits. The bivariate analysis (Table 1) shows that the majority (75.5%) of women aged 20–24 went for at least four antenatal visits. A higher proportion of women aged 35 and above went for more than four antenatal visits. Age is significantly associated with the use of antenatal services (P < 0.01). The majority of women who reported that they were married (60.0%) went for at least four antenatal visits, while (42.0%) of women who were not married went for five and more antenatal visits. A large proportion (74.4%) of women who reported to that they were self-employed went for the recommended number of antenatal visits (at least four). Similarly, women who reported that they did not find it difficult to get money to go to hospital had high proportions of those who went for antenatal visits (60.9%). Regarding means of travelling to hospital, 66.7% of women who reported that they use a private transportation went for at least four antenatal visits, while the majority (61.1%) of those who reported that they walked to hospital went for more than four antenatal visits.

The multivariate analysis revealed that women aged 35–39 and 40 (and above) were 5.5 and 5.3 times more likely to use antenatal services than those aged 15–19 respectively. The age cohorts, 35–39 and 40+ were significantly associated with use of antenatal visits (P < 0.05). Women who reported that they were not married were 1.5 times more likely to use antenatal services compared with those who were married. Regarding maternal education, women who reported that they had attained secondary-level education were 2.9 times more likely to use antenatal services compared with women who have no education. Women who use private transportation to travel to hospital were 0.2 times less likely to use antenatal services than those who walk to hospital. Furthermore, women who reported that they did not have any cultural factors which prevent them from using maternal health services were 1.7 times more likely to use antenatal services compared with those who indicated that cultural factors prevent them from using maternal health services. Women who did not find the maternal health information useful and those who had no prior knowledge of maternal health services offered were less likely to use antenatal services.

### Qualitative analysis

#### Determinants of maternal health care use: perspectives of health care professionals

Staff shortages became a major theme in the analysis when it came to trying to understand the challenges encountered by health professionals while working within the maternal health unit. Shortages of health care staff can have serious implications for the utilization of maternal health services, and it serves as a barrier which hinders the use of maternal health services. With regard to staff shortages, one nurse stated that:

*“The waiting time is long”*

It has been found that shortages of health professionals and longer waiting times reduce the satisfaction of patients with the health-related services they receive [21] – what is more is that this then leads to a decline in the utilization of health services. For instance, this can also have an influence on patients not seeing the need to seek maternal health care services; whereby they will argue that ‘why go to the hospital if you will not receive the adequate care that you need?’

One health professional argued that mothers who attend antenatal care services face a number of challenges (problems); these relate to the issue of having to travel long distances, financial problems, and the attitude of health professionals.

*Most mothers – and those who are expecting, face “financial problems, [whereby] they won’t come on time here when referred and they will tell us that they didn’t have money to come here”*

Digressing on time (allocated for appointments) and not seeking the available help can also be viewed in relation to the attitude of health professionals. This was also echoed by a doctor who stated that:

*“Sometimes the attitude from the health care workers hinders the satisfaction and use of maternal health services”*

One could argue that, due to the shortage of health professionals there is a burden placed on the available staff to attend to a large number of women seeking maternal health services. This then has an implication on the type and quality of service offered, whereby some staff members will often take their frustrations out onto the patients. Moreover, the attitude of health professionals towards their patients is often driven by their stigma in relation to some health problems and certain social circumstances. For instance, with regards to teenage pregnancy and HIV, there seems to be a general stigma among health professionals. The doctors interviewed argued that teenage pregnancy and HIV were among the challenges they have encountered while working within the maternal health unit; moreover, one doctor argued that, with regards to the treatment received by pregnant teenagers and those who are HIV-positive:

*“The clinic people [staff], they just chase them away and send them without helping them which is really a problem and the other clinics the still chasing them for being HIV positive to the hospital which… is not a good thing because we’ve to treat people the same”*

The health professionals noted a number of other reasons which prevent most women from utilizing maternal health services available at the hospital. The health professionals noted ‘*lack of knowledge’* as one of the factors that contribute to women not using maternal health services. Most women are not even aware that they should be going for antenatal services when they are pregnant. One maternal health care specialist argued that:

*“Lack of knowledge is the most contributing factor to the low use of maternal health services. They [patients] are not aware that they should be coming to maternal health care [sessions] when they are pregnant; [and] they don’t even know what maternal health care is”*

Besides not being aware of maternal health services offered at the hospital, most women *“are not being referred”* to antenatal care by their nurses and doctors. This has an impact on the frequency with which women utilise maternal health services. If women are not being referred to these services then they will not be able to utilise such services. One maternal health care specialist stated that pregnant women are:

*“Only referred for maternal health care services when there is a problem” or when there is a complication, and these problems could be prevented if referrals could be done at initial contact”*

## Discussion

The results suggest that there are several determinants of maternal health care utilization in the province. These determinants have an influence on the frequency with which women use the available maternal health services. Women who have had bad experiences tend to utilize maternal health services less frequently than those who have had good experiences. For instance, a woman who must wait for a long time before being attended (perhaps even going home unattended) might see no need to return to the clinic, unlike the one that received attention within a reasonable amount of time. Moreover, if a patient receives poor treatment, then that patient will view the health system in a negative light. Use of maternal services can be influenced by the attitude one has about the services offered. If a woman has a ‘*willing to learn*’ or accepting attitude then there are better chances that she will make use of the services offered. Conversely, financial problems were also noted as one of the most significant factors that influence the use of maternal health services. Women who did not find it difficult to get money to get to hospital were more likely to access maternal health services than those who found it difficult to get money to travel to hospital.

Previous studies have found that women in rural areas, those who have to travel long distances, and those who have received poor quality of care tend to use maternal health services less [6, 9, 16, 19, 20]. Therefore, having to travel long distances to seek maternal health care can be a barrier to complete use of maternal health care services, especially in rural areas where there is a low socio-economic status among such populations [19, 22]. In such situations, financial problems also become a barrier to the frequency with which these women seek medical attention – whereby pregnant women might not be able to afford transportation that will take them to hospital. Regarding this trend in the study area, a health professional noted:

*“Most of the women who go to seek maternal health services have to travel long distances, since most of them stay in rural areas, and sometimes they do not have enough money to get to the clinics or hospitals”*

Besides financial problems, lack of information also poses a serious hindrance to the use of maternal health services. The quantitative results show that most women (37.7%) who reported that they had knowledge of the maternal health services offered accessed more maternal health services than those who did not know about the services offered. The study has indicated that most women are not aware of available maternal health services, and this lack of awareness leads to minimal use of these services. In the study area, health professionals do not often refer pregnant women for antenatal services, and when those women have delivered, they are not referred for postnatal services, and this contributes to the less frequent use of maternal health services. Sometimes, women do not go back to clinics for check-ups after they have delivered – and they see no need to do so, unless certain complications arise. To support this claim, the quantitative analysis showed that women who did not find the information provided about maternal health services useful were less likely to access and use these services than those who found the information provided useful.

Cultural factors also constitute an important when it comes to the use of maternal health care services. In South Africa, culture is an important concept that influences the way people live, as well as their belief systems. For instance, there are women that believe in utilizing traditional birth attendants, rather than seeking professional health care, due to their cultural beliefs; these women tend to opt for home-based deliveries, assisted by traditional birth attendants, rather than going to a hospital or clinic [23]. Access to medical aid is another important factor related to the use maternal health services. Women who reported that they did not have access to medical aid were less likely to access maternal health services compared with those who had medical aid. Generally, people who have access to medical aid tend to use private health facilities rather than public health facilities. A reason for this could be that due to the kind of treatment women generally receive in public hospitals, they opt for a medical aid which gives them access to private health facilities; as discussed, public hospitals are often short-staffed and as such, adequate care of patients is often overlooked.

## Conclusion

The analysis has shown that women in the study area face a lot of challenges regarding access and use of maternal health services. Only a small percentage of women (35.2%) are accessing maternal health services. The study findings suggest that there are several barriers and determinants associated with use of maternal health services in the study area. These include lack of knowledge about maternal health services and poor quality of services among others. The results also point to the status of the South African public health system. The responses from the health professionals point to the fact that the public health system is not properly functioning. Many hospitals (especially those in townships and rural areas) tend to be neglected and usually do not have adequate staff to render health services to scores of people who seek such services. Health care use is determined by the way in which health professionals treat patients. There is still stigma associated with certain health conditions (as well as social situations). For example, there are still health professionals who refuse to assist (or often maltreat) young girls who fall pregnant in their teenage years; moreover, these professionals even go as far as to mistreat pregnant women who are HIV positive.

A lot can be done to improve the accessibility and utilization of maternal health services. One such improvement would be to ensure that information relating to the importance (and use) of maternal health services is properly disseminated so that more people can have knowledge about such services. A number of other strategies could play a big role in campaigning for better access and use of maternal health services, especially in rural areas. These strategies could include (a) the inclusion of the media in terms of broadcasting information relating to maternal health services and the importance of such services, (b) educational programmes aimed at enhancing the literacy skills of women (especially in rural areas), (c) implementing better policies that are aimed at shaping the livelihoods of women, and (d) implementing better maternal health care delivery in rural areas as well as other areas where there is a lack of delivery.

### Limitations

The sample size (267 participants; and 6 interview participants) does not represent a complete picture of the status of maternal health care services in South Africa, but this sample does give an indication of the situation regarding these services within the Primary Health Care sector. Therefore, the data is not representative of the whole South African population, but gives us an idea of the way maternal health services are accessed and used in this area within the Eastern Cape Province.

### Ethical considerations

Ethical issues (including plagiarism, informed consent, misconduct, data fabrication and/or falsification, double publication and/or submission, redundancy, etc.) have been completely observed by the authors. This study is immensely useful for the policy makers and planners.

## Authors’ information

Mr. Mluleki Tsawe is a Masters student in Department of Statistics & Population Studies at the University of the Western Cape. Professor A Sathiya Susuman has an MA, MPhil in Population Studies and a PhD in Demography. He has specialized in the social science research area of demographic analysis and reproductive health. His specific research area is fertility, mortality, gender, reproductive health and public health. He has published several articles in reputed journals.
